# Assessment of Margins in Transoral Laser and Robotic Surgery

**DOI:** 10.5041/RMMJ.10150

**Published:** 2014-04-28

**Authors:** Yaniv Hamzany, Daniel Brasnu, Thomas Shpitzer, Jacob Shvero

**Affiliations:** 1 Department of Otorhinolaryngology—Head and Neck Surgery, Rabin Medical Center, Petach Tikva and Sackler Faculty of Medicine, Tel Aviv University, Tel Aviv, Israel and; 2 Department of Otorhinolaryngology—Head Neck Surgery, Université Paris Descartes, Sorbonne Paris Cité, Hôpital Européen Georges Pompidou, Assistance Publique—Hôpitaux de Paris, Paris, France

**Keywords:** Cancer, glottis, laser, margin, robot, transoral

## Abstract

The growing practice of endoscopic surgery has changed the therapeutic management of selected head and neck cancers. Although a negative surgical margin in resection of neoplasm is the most important surgical principle in oncologic surgery, controversies exist regarding assessment and interpretation of the status of margin resection. The aim of this review was to summarize the literature considering the assessment and feasibility of negative margins in transoral laser microsurgery (TLM) and transoral robotic surgery (TORS). Free margin status is being approached differently in vocal cord cancer (1–2 mm) compared with other sites in the upper aerodigestive tract (2–5 mm). Exposure, orientation of the pathological specimen, and co-operation with the pathologist are crucial principles needed to be followed in transoral surgery. Piecemeal resection to better expose deep tumor involvement and biopsies taken from surgical margins surrounding site of resection can improve margin assessment. High rates of negative surgical margins can be achieved with TLM and TORS. Adjuvant treatment decision should take into consideration also the surgeon’s judgment with regard to the completeness of tumor resection.

## INTRODUCTION

A negative surgical margin in resection of neoplasm is well recognized as the most important surgical principle in oncologic surgery.^1^ In order to achieve complete surgical excision the resection should include a cuff of healthy tissue surrounding the neoplasm in all three dimensions. Lack of adequate negative margins can doom the patients to repeat surgery or adjuvant oncologic treatment. Despite that, there is no unanimity about the size and the method to assess the healthy tissue surrounding the tumor. Ambiguous terms such as “close margin” or “inconclusive” further contribute to the unclarity of margin evaluation and decision-making.

The search of surgical procedures that better preserve function and quality of life, parallel to technological progress, has led to the development of endoscopic approaches in head and neck surgical oncology. Strong described the first use of endoscopic CO_2_ laser resection of glottic cancer in 1975.^2^ With further development over the next decades, the technique became one of the mainstay treatments for early laryngeal cancer.^3^–^6^ Transoral robotic surgery (TORS) for the resection of supraglottic cancer was introduced in 2007 by Weinstein et al.^7^ overcoming some of the limitations concerning visualization, maneuvering, and accessibility in transoral laser microsurgery (TLM).

The growing practice of endoscopic surgeries resulted in a change in the therapeutic management of selected head and neck cancers, replacing the external approach in early stages.^5^

The aim of this review was to summarize the literature considering the assessment and feasibility of negative margins in transoral laser and robotic surgery.

## BASIC PRINCIPLES IN ENDOSCOPIC SURGERY

Transoral laser microsurgery is minimally invasive and is performed under direct suspension laryngoscopy with an operating microscope that grants the surgeon a high-power magnification of vision, therefore a superior detailed quality compared to that obtained by external approach. In TORS one of the arms holds a high-definition endoscopic camera, enabling an excellent three-dimensional magnified vision which can be moved during the surgery. However, the tactile feedback in endoscopic operation is limited or not possible; therefore assessment of tumor penetration is hampered. In order to overcome its limitations, and fully utilize its advantages, transoral surgery dictates some changes and emphasizes different principles during the operation.

### Exposure

Obtaining good exposure of the lesion is an important principle in surgical oncology; it is a key parameter to the success of the endoscopic procedure. Several studies have found the surgeon’s judgment of complete resection of glottic cancer in TLM to be superior to violated margins in the histopathology report.^8^–^11^ The surgeon’s assessment of the neoplasm borders, based on the excellent view, necessitates as clear and complete a view of the lesion as possible. From setting up the patient in the correct position, through using the different kinds of laryngoscopes or retractors, setting up the microscope and robotic arms in the limited transoral field of surgery, although time-consuming, is part of the transoral surgery. Depending on the site of the tumor, especially in transoral laser cordectomy, exposure can necessitate resection of obscuring tissue such as false vocal cord or petiole of the epiglottis. Large tumors can be transected and excised by a multiblock approach (piecemeal), revealing depth of tumor penetration, with clear visualization of tumor margins and without hampering oncologic results.

### Orientation

Preserving the three-dimensional orientation of the tissue during the resection can be quite difficult, especially in bulky tumors involving multiple sites. In order to avoid unnecessary distractions from the operative field during surgery the nursing staff in the operation room must be familiar with the endoscopic equipment and the surgeon’s preferences. Marking designate borders with clips or ink during the resection or immediately after the tumor has been removed can add substantially in avoiding disorientation of the specimen. While piecemeal resection helps to excise a large-volume tumor and determine its depth of invasion, it also adds to the complexity of margin evaluation. Using different ink colors helps distinguish true oncologic margin from intraoperative non-margin tissue cut. Documenting the resection by translating the three-dimensional resection to a two-dimensional diagram can be challenging; however, it is very helpful in clarifying the resection.

### Co-operation with the Pathologist

The importance of good communication and understanding with the pathologist cannot be over-stressed. A schema including labels to the anatomic and specimen sub-sites, as well as pinning the specimen on a corkboard with designation of the adjacent tissues can significantly help the pathologist in understanding the relations of the specimen to adjacent tissues in space. Handing off the specimen personally to the pathologist can be the best way to elucidate the anatomy while emphasizing the important zones for gross preliminary assessment. Information on close or positive margin can be suggested by the pathologist, with the possibility to return to the operating room and expand the resection if needed.

Margin status is one of the most influential parameters on decision-making when discussing adjuvant treatment. Margins are commonly measured from the tumor invasive front to the nearest surgical resection edge. While free margins or involvement of the tumor in the surgical cut is mostly obvious, there is controversy on the crucial issue of the distance required between the carcinoma and the surgical cut. What is the definition of close margin necessitating further consideration? Since every region in head and neck has its own characteristics in terms of lymphatic drainage, vascular supply, or anatomic barrier (e.g. fascia, perichondrium, periosteum), using the same definition of close margin for all regions can be inappropriate.

The National Comprehensive Cancer Network (NCCN), American Society of Clinical Oncology (ASCO), and European Oncology Institute (IEO) guidelines define a close margin as ≤5 mm without any sub-site distinction. In a survey of the American Head and Neck Society, regarding the definition of margins, the most common response was that a clear margin was >5 mm on microscopic evaluation.^12^ Hinni et al.^13^ in their comprehensive review on surgical margins in head and neck reported that most studies use a margin distance of ≥5 mm to define margin adequacy, with the exception of glottic cancer in which there is a long-standing consensus that resection margins may be as limited as 1 to 2 mm and still be considered adequate. Another review studied the question of what a close margin is in head and neck squamous cell carcinoma.^14^ The conclusion was that in vocal cord surgery a close margin could be considered as ≤1 mm, in the larynx as ≤5 mm, in the oral cavity as ≤4 mm, and in the oropharynx as ≤5 mm. For this reason assessment of margins is being approached differently in vocal cord cancer compared with other sites in the upper aerodigestive tract.

## ASSESSMENT OF MARGINS IN ENDOSCOPIC SURGERY—GLOTTIC CANCER

When treating early glottis cancer with TLM, a 1–2 mm free margin from the tumor line is sufficient to guarantee a complete resection.^11^,^15^,^16^ In order to obtain good functional results the resection is tailored to the clinical appearance of the tumor, sparing as much tissue as possible of the vocal cord. It is not uncommon therefore to have close or positive margins on permanent histopathologic analysis of the main specimens.

Several studies that have addressed the impact of margins status on local control in TLM for glottic cancer have provided contrasting results (Table 1).^8^–^11^,^17^–^20^ While Peretti,^19^ Ansarin,^18^ and Crespo et al.^20^ have suggested a worse outcome in patients with close or positive margins, Brondbo,^8^ Hartl,^9^ and Michel et al.^10^ have published contradictory findings. The rate of inadequate or positive margins on final pathology ranged from 6% to 50%. Reresection was performed only in part of the patients with close or positive margins, while adopting a policy of close follow up in the rest. In cases of re-resection, the rate of positive pathology was 0%–14%. In all the studies the rate of local recurrence was higher in cases of inadequate or close margins in first resection, compared to patients with negative margins, 3%–37.5% and 0%–9%, respectively. However, statistically significant differences were reported only in three studies. The rate of initial local control was 84%–96%.

**Table 1.** Studies Addressing the Impact of Margins Status on Local Control in TLM for Glottic Cancer

**Author**	**Patients**	**T Stage**	**Adequate Margin Definition**	**Use of Frozen Section**	**Inadequate or Positive Margins on Final Pathology**	**Further Treatment for Inadequate or Positive Margins (Number of Patients; Treatment)**	**Rate of Positive Pathology on Second Resection**	**Rate of Local Recurrence in Inadequate or Positive Margins**	**Rate of Local Recurrence in Negative Margins**	**Significant Impact of Margins Status on Local Control**	**Initial Local Control Rate**
Fang^17^ (2013)	75	T1, T2	NA	yes	I: 10 (10%) P: 28 (40%)	none		18%	3%	no	84%
Michel^10^ (2011)	64	T1	1–2 mm	no	24 (37.5%)	10; re-resection	10%	17%	5%	no	91%
Peretti^19^ (2010)	595	Tis-T3	>1 mm	no	300 (50%)	71; re-resection	14%	7%–15%	6%	yes	85%
Ansain^18^ (2009)	274	Tis-T1	>1 mm	no	I: 40 (15%) P: 54 (20%)	28; RT 36; re-resection	8%	14%	2%	yes	91%
Hartl^9^ (2008)	79	Tis-T1	>2 mm	no	I: 20 (25%) P: 5 (6%)	3; resection	0	24%	9%	no	96%
Manola^11^ (2008)	31	T1	1–2 mm	no	3 (10%)	3; resection	0	3%	0	no	95%
Brondbo^8^ (2007)	171	T1	1–2 mm	no	62 (36%)	none		14.5%	4%	no	91%
Crespo^20^ (2006)	40	T1, T2	NA	yes	P: 8 (20%)	2; RT		37.5%	0	yes	93%

I, inadequate; NA, data not available; P, Positive; RT, radiation therapy; TLM, transoral laser microsurgery.

Several factors can contribute to the controversy of interpretation and impact of positive margin in TLM, including small specimen size, tissue retraction, and thermal effects induced by the laser. Tissue fixation induces a shrinking of >30% and can therefore influence assessment of margins on final pathology.^21^ Interpretation of the pathology report should take into account that peripheral coagulation is about 0.3–0.5 mm wide, which increases the true resection margin by about that much as compared to the pathologist’s measurements.^8^,^9^ Furthermore, cells with genetic alterations which are not yet histologically visible may be present in the non-neoplastic tissue close to the tumor.^22^,^23^ Their subsequent development would lead to an apparent local recurrence that is simply the expression of the natural history of pre-existing lesions.

Special care should be taken in glottic cancer with involvement of the anterior commissure or deep surgical margin. Several articles have reported on lower local control rate in glottic cancer when involvement of the anterior commissure was found.^24^–^29^ The difficulty in adequate exposure of the anterior commissure using conventional laryngoscope can contribute to this result, stressing the importance of fully exposing this site during TLM by the use of larger and better designed laryngoscopes and by resection of supraglottic tissue as necessary.^28^,^30^ Anatomic constraints and hampered visibility may limit the surgeon’s ability to achieve adequate deep surgical margins. Peretti et al.,^19^ who evaluated the impact of superficial and deep positive margins in 595 patients treated with TLM for glottic cancer, found low impact of superficial positive margins on local control compared to deep infiltration (93% versus 85%). Transection of the tumor can give the surgeon a much better assessment of the depth dimension and clear visualization of the deep margin during surgery.^31^

In order to ameliorate margin assessment different techniques have been studied.

### Frozen Section

One of the valuable techniques is intraoperative resection margin evaluation by using a frozen section analysis for biopsy taken from the cut border of tissue remaining in the patient. Remacle et al.^32^ found frozen section to be reliable with 95% of the results accurate and stressed the possibility immediately to enlarge cordectomy to obtain clear margin. Fang et al.^17^ reported that the status of the initial frozen-section margin analysis is a robust predictor of survival. In patients who had involvement by malignancy of the initial resection margin on frozen section, there was a statically significant increased rate of recurrent disease within the first year regardless of eventually achieving clear margins during the initial surgery.

When using frozen section, one has to be familiar with its drawbacks. The reliability of a margin verdict using small fragments taken from the cut border of tissue remaining in the patient depends on the surgeon’s precision and the pathologist’s experience. Insufficient biopsy material or biopsy taken in between neoplastic cells can produce false negative results. Postoperative or post-radiotherapy patients can have granulation tissue, inflammatory infiltrate, or post-irradiation cell changes, making diagnosis more difficult. Moreover, use of multiple frozen sections for margin control, after the tumor has been removed, has intrinsic discrepancy with the phonomicrosurgical approach to the treatment of vocal cord cancer.

### Second-look Laryngoscopy

In order to avoid unrecognized positive margin, due to technical difficulties of anatomical orientation as well as cauterization artifacts associated with laser carbonization, and subsequently untreated residual carcinoma, second-look laryngoscopy management has been suggested. Roh et al.^24^ have evaluated the efficacy of second-look laryngoscopy in patients with glottic cancer involving the anterior commissure. They concluded that it is unclear whether routine second-look laryngoscopy is necessary in detecting tumor recurrence and suggested that it should be performed at a time later than 3 months after first surgery. Preuss et al.^33^ stressed the efficiency of a second-look procedure in detection of recurrent disease at a very early stage, also suggesting that the interval between the first surgery and the second-look laryngoscopy should be longer than 10 weeks.

The benefits of a routine practice of second-look laryngoscopy should be evaluated against the additional stress, risks, and high cost of surgery with general anesthesia.^32^

### Optical and Molecular Techniques

Over the past two decades several optical imaging technologies have been used in the operating room in order to improve the ability to identify tumor margin *in vivo* and *in situ* to guide surgical excision. This concept is particularly important for lesions on the vocal cords where conservation of the delicate superficial lamina propria is crucial for preservation of voice quality.

Andrea et al. were the first to use contact endoscopy in the diagnosis of laryngeal disease in 1995.^34^ By using a magnifying endoscope placed in direct contact with the mucosal surface, images at ×60 or ×150 magnification of the superficial layers of the vocal cord epithelium are obtained.^35^ In the diagnosis of malignant lesions sensitivity and specificity rates of 80% and 100%, respectively, have been reported.^36^ An important limitation of contact endoscopy is its inability to give clear images of cells beyond the most superficial layers of the epithelium, meaning the basement membrane; therefore distinction between cis and invasive carcinoma is prevented.^37^

Hughes et al. reviewed the efficacy of different optical and molecular techniques to identify tumor margins within the larynx.^37^ They conclude that further research and randomized clinical trials are required to validate these techniques and establish their benefit to patients.

## ASSESSMENT OF MARGINS IN ENDOSCOPIC SURGERY—NON-GLOTTIC CANCER

For external approaches, recommendations regarding safety margins in the oropharynx, hypopharynx, and supraglottic most commonly range from 5 mm to a few centimeters, depending on tumor site and surgeon. TLM aims to preserve as much healthy tissue as possible in order for function to be maintained and to enable early recovery, and although wider free margins than in the vocal cords are commonly accepted, a large distance as in external approach is uncommon.

In 2011, a National Cancer Institute Head and Neck Cancer Steering Committee Clinical Trials Planning Meeting published recommendations for transoral resection of pharyngeal cancer.^38^ Recommendations included use of frozen section to guide resection until margins are tumor-free circumferentially around the tumor. On final pathology report margins will be recorded as either “clear” (negative) or “involved” (positive). “Close” margins can be recorded, but will not influence the “risk” status of the tumor and subsequent treatment. Blanch et al. stressed that, in patients who have been diagnosed with pharyngo-laryngeal cancer, TLM can generate situations where it is difficult to define the boundary between tumor-free tissue and tumor-affected tissue.^39^ When tumor cells were found at less than 2 mm from the margin, when carbonization impaired margin assessment, or when no final pathology could be obtained (thyroid cartilage has been reached), this was considered an uncertain margin. Their results showed that patients with positive or uncertain margins were more likely to have relapsed than patients with negative margins, concluding that status of tumor margins is an independent prognostic factor influencing local control. However, Jackel et al.^40^ concluded differently after analyzing the results of one of the biggest series of patients with upper aerodigestive tract cancer treated with TLM. They found that it is the neoplastic cells in the revision specimen that count as a prognostic factor for poor local control, rather than a positive margin in the initial specimen. Hinni et al.^41^ used a technique they named margin mapping: careful, microscopically driven piecemeal tumor resection, where the inking and preparation of the specimen are done in the operating room by the operating surgeon in close consultation with the pathologist, in TLM for tonsil cancer. Their conclusion challenged the concept that a margin of 5 mm should be obtained to prevent local recurrence, also suggesting that such a margin is not supported by local anatomy.

TORS offers the possibility of improved visualization and better accessibility over TLM. The daVinci Surgical System (Intuitive Surgical^®^ Inc., Sunnyvale, CA, USA) consists of a surgeon’s console and a surgical cart comprising two laterally placed instrument arms and a centrally located endoscopic arm holding the three-dimensional camera. During TORS the surgeon has real-time and direct control of instrument movement, with the possibility to use open surgical techniques via the console. These high-level capabilities make the daVinci robot more suitable to perform oncologic resections and the surgeon to perform more complex operations.

Assessment of margins, as described in the first reports in the literature, uses the same principles of TLM with good co-operation between the surgeon and the pathologist and biopsies taken for frozen section analysis as needed.^42^–^44^ Table 2 summarizes publications of up-to-date series of TORS for upper aerodigestive tract cancer with assessment of surgical margins.^45^–^55^ Rates of inadequate or positive surgical margins on pathology report were 0%–33% with local control rates of 91%–100%.

**Table 2.** Up-to-date Series of TORS for Upper Aerodigestive Tract Cancer with Assessment of Surgical Margins.

**Author**	**Year**	**Site (all T stages unless otherwise indicated)**	**Patients**	**Close Margin Definition**	**Use of Frozen Section**	**Inadequate or Positive Margins on Final Pathology**	**Local Control**
Boudreaux^45^	2009	Oral cavity, oropharynx, hypopharynx, supraglottic	29	NA	yes	0	NA
Weinstein^46^	2010	Oropharynx	47	≤2 mm	yes	1 (2%)	98%
White^47^	2010	Oral cavity, oropharynx, supraglottic	89	NA	yes	3 (3%)	97%
Hurtuk^48^	2011	Oropharynx, hypopharynx, larynx (1–3)	54	≤2 mm	yes	4 (7%)	98%
Lawson^49^	2011	Oral cavity, oropharynx, hypopharynx, supraglottic (1–3)	24	NA	yes	0	92%
Hans^50^	2011	Oropharynx, hypopharynx, supraglottic	23	NA	NA	1 (4%)	100%
Aubry^51^	2011	Oropharynx, hypopharynx, supraglottic (1–3)	17	NA	yes	2 (12%)	100%
Genden^52^	2011	Oropharynx, hypopharynx, larynx (1–3)	30	NA	yes	10 (33%)	91%
Park^53^	2012	Hypopharyngeal	23	≤5 mm	NA	2 (9%)	100%
Park^54^	2013	Oropharynx	39	≤5 mm	NA	2 (5%)	95%
Park^55^	2013	Supraglottic (1–3)	16	NA	NA	2 (12%)	100%

* Mean follow-up time <12 months.

TORS, transoral robotic surgery; NA, data not available.

Weinstein et al. found TORS may offer local control rates for oropharyngeal cancer similar to if not better than those seen with TLM, suggesting greater confidence in the surgical margin assessment seen with TORS en-bloc resection that lends itself to potentially more accurate pathologic evaluation.^56^ This result was not in concordance with Ansarin et al. who evaluated TORS versus TLM for resection of supraglottic cancer.^57^ Although en-bloc resection was reported to be easier with TORS, a higher proportion of positive resection margins was found with TORS than with TLM, 40% and 20%, respectively.

## CONCLUSION

- Exposure, orientation, and co-operation with the pathologist are crucial principles needed to be followed in transoral surgery for success of margins assessment.
- Resection should be done with clear microscopic margins on pathologic report of 1–2 mm for glottic cancer and 2–5 mm for other non-glottic cancer. Piecemeal resection can be done, as needed, to better expose deep tumor involvement.
- Preservation of histological specimen orientation should be done by pinning the specimen on a corkboard with designation of the adjacent tissue and inking surgical margins as needed.
- A schema including labels to the specimens and adjacent anatomic sub-sites can be very useful if expansion of margin is needed.
- Biopsies taken from surgical margins in critical sites surrounding site of resection, especially in deep borders, either for frozen section or final pathology, can lead to significant improvement of margin assessment.

Although high rates of negative surgical margins can be achieved with TLM and TORS, decision-making on the need for adjuvant treatment should take into consideration not only the pathology report but also other important parameters during surgery such as the feasibility of exposure and the surgeon’s judgment with regard to the completeness of excision. In order to have better evaluation and understanding of oncologic results it is necessary to form a consensus on how to assess and define surgical margins in transoral endoscopic surgery.

## Abbreviations:

TLM: transoral laser microsurgery
TORS: transoral robotic surgery.
